# Pumping Iron for Tuberculosis Diagnostics

**DOI:** 10.1021/acscentsci.5c00133

**Published:** 2025-02-11

**Authors:** Abdulai Zigli, Benjamin M. Swarts

**Affiliations:** † Department of Chemistry and Biochemistry, Central Michigan University, Mount Pleasant, Michigan 48859, United States; ‡ Biochemistry, Cell, and Molecular Biology Graduate Programs, Central Michigan University, Mount Pleasant, Michigan 48859, United States

Tuberculosis (TB) is a major and urgent global health priority, as there are approximately 10 million TB cases and over 1 million TB deaths every year, making it the leading cause of death worldwide by infectious disease. A significant challenge in controlling TB is early and accurate diagnosis of the disease. The gold standard for TB diagnosis is the culture of *Mycobacterium tuberculosis* (Mtb), the causative agent of TB. However, due to the slow growth rate of Mtb, culture tests can take over a month to deliver a diagnosis. Although advanced molecular methods such as nucleic acid amplification and whole-genome sequencing are becoming more widely adopted, they remain equipment-intensive and relatively expensive. Fast, simple, and inexpensive methods are needed to detect Mtb, particularly in low-resource settings. Historically, this need has been met by sputum smear microscopy utilizing Ziehl–Neelsen or auramine–rhodamine staining to directly visualize Mtb in patient samples. However, the drawbacks of traditional TB staining methods, such as limited sensitivity, specificity, and/or convenience, have prompted significant efforts to develop novel fluorescent probes for improved detection of Mtb.^1^

Several groups have recently reported fluorogenic probes for mycobacteria, which undergo dark-to-light fluorescence activation via mycobacteria-specific mechanisms. Selected examples include probes that are activated by mycobacterial trehalose metabolism,^2,3^ β-lactamase,^4^ nitroreductase,^5,6^ or protease^7^ activity. Still, many of these tools have limitations (e.g., reagent concentrations, rates of labeling) and require additional research to explore their clinical viability, motivating the development of alternative approaches.

**Figure 1:**
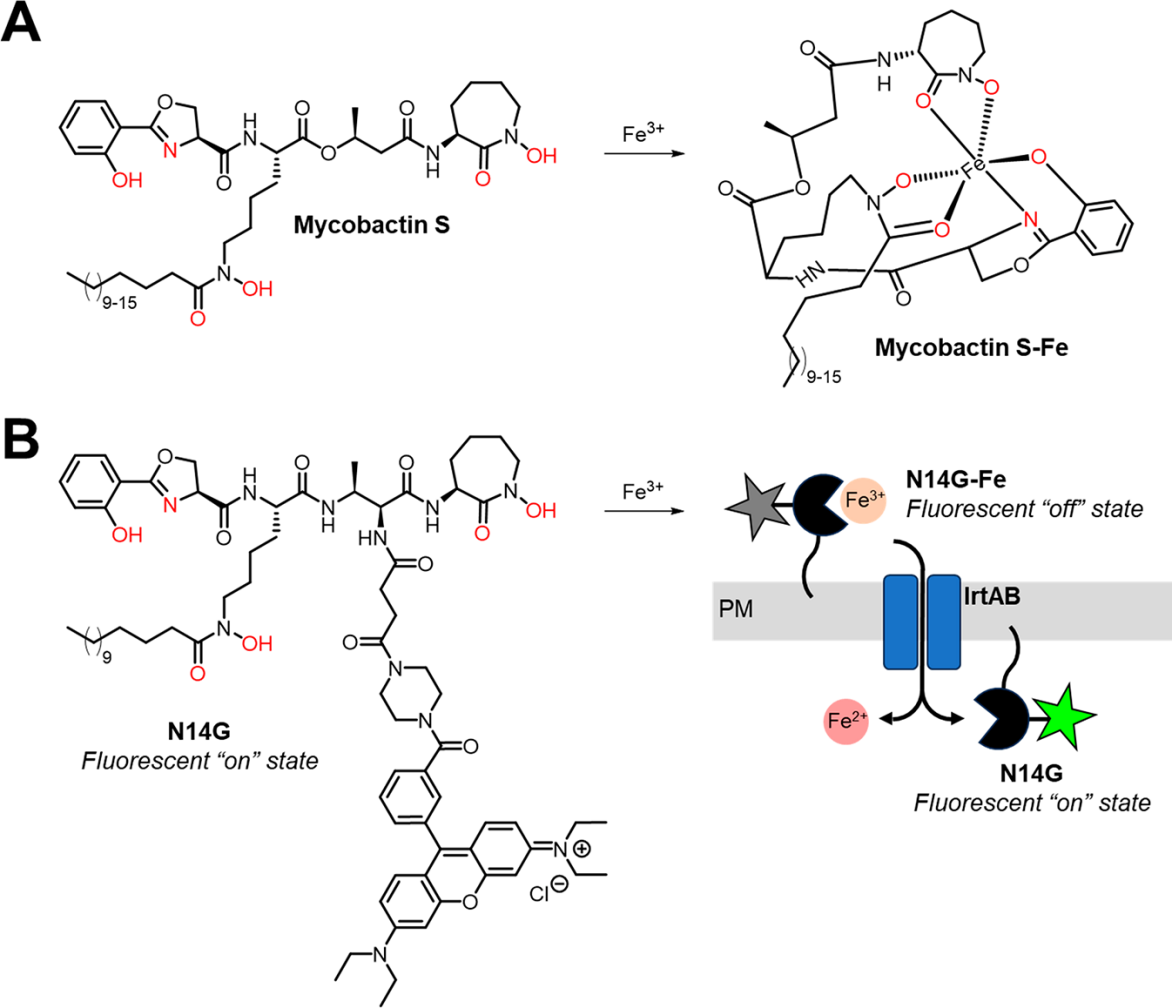
(A) Structure of a representative mycobactin, mycobactin S, in free and Fe^3+^-bound form. (B) Structure of mycobactin–fluorophore conjugate N14G and proposed mechanism of fluorogenic detection of mycobacteria. PM, plasma membrane.

Mtb is an obligate human pathogen that has evolved to scavenge iron—an essential nutrient—from the host through mycobactin synthesis, secretion, and reuptake in iron-chelated form.^8^ To allow this function, mycobactins possess a common core structure exhibiting three hydroxy-amide/oxazoline moieties that bind Fe^3+^ in a hexadentate manner (Figure 1A). After Fe^3+^ binding in the extracellular environment, iron-chelated mycobactin is actively imported into Mtb via the plasma-membrane-associated ATP-binding cassette (ABC) transporter IrtAB, which proceeds with concomitant intracellular reduction of Fe^3+^ to Fe^2+^ by the siderophore interaction domain and, ultimately, release of Fe^2+^ and free mycobactin. Seeking to hijack the IrtAB iron scavenging pathway to create a fluorogenic probe for Mtb detection, Liu and co-workers designed and synthesized several fluorophore-conjugated mycobactin derivatives, including the rhodamine–mycobactin conjugate N14G (Figure 1B). Promisingly, free N14G was found to have >25-fold higher fluorescence than the Fe^3+^-chelated form, N14G-Fe, a quenching effect that the authors propose to be due to the paramagnetic and electronic properties of Fe^3+^. This observation suggested that upon IrtAB-mediated uptake of N14G-Fe by mycobacterial cells, the reduction of Fe^3+^ to Fe^2+^ would release free N14G and trigger fluorescence turn on. Consistently, when mycobacterial cells were incubated with N14G-Fe, dramatic fluorescence enhancements were observed, even when direct measurement of probe-treated, unwashed cells was conducted.

A potential limitation of previously reported fluorogenic probes is that they are used in the micromolar concentration range, which could reduce assay sensitivity and strain resources for difficult-to-synthesize probes. The authors demonstrate here that the N14G mycobactin probe is exceptionally sensitive, detecting various mycobacterial species when administered in the low nanomolar range for short durations. Mtb was robustly detected by N14G when administered at a 100 nM concentration for only 10 min, and the limit of detection for N14G was found to be an order of magnitude better than that of a reported^5^ nitroreductase-activated fluorogenic probe. The mycobacteria specificity of N14G also appears to be promising, owing to the mycobactin–IrtAB system being unique to mycobacteria. N14G labeled the model organism *Mycobacterium smegmatis* in an IrtAB-dependent manner, showed less or no labeling of nonmycobacterial species, and successfully detected Mtb in processed sputum samples from TB patients. N14G labeling of mycobacteria was also substantially diminished by heat-killing or antibiotic treatment, suggesting the probe can be used to report on bacterial viability, a property which can be leveraged in drug-susceptibility assays needed for diagnosing drug-resistant TB.^3,6^

Although N14G labeling was shown to be dependent on plasma-membrane-associated IrtAB, it is not yet known how the probe traverses the large and complex mycobacterial cell wall, including the outer membrane. In addition, the observations of (i) some IrtAB-independent labeling by N14G, (ii) equal labeling of mycobacteria by N14G and a chelation-incompetent N14G analogue, and (iii) modest N14G labeling of some nonmycobacterial organisms together suggest the possibility of some nonspecific incorporation and/or additional transporters that uptake the probe. Shedding additional light on the probes’ incorporation mechanism(s), structural requirements, and specificity for Mtb versus other cell types (e.g., mammalian cells and nontuberculous mycobacteria) will provide a more comprehensive understanding of their uses and limitations. Several potential applications of this new class of fluorescent probes are immediately apparent. Mycobactin–fluorophore conjugates can be used as tools to probe mycobacterial iron acquisition pathways, which have gained attention in antitubercular drug development.^8^ The probes represent a new entry into—and tool to facilitate the development of—siderophore-based Trojan horse strategies to deliver chemical cargo to bacteria, a variety of which have previously been developed, including mycobactin–drug conjugates.^9^ Finally, and attractively from the clinical standpoint, mycobactin-based fluorogenic probes have potential as sensitive detection reagents to aid in the rapid diagnosis of TB.
